# Supplementary material for: Defects in nephrogenesis result in an expansion of the Foxd1+ stromal progenitor population
Source: Development. 2026 Jan 7;153(1):dev204964. doi: 10.1242/dev.204964 (PMC12848570; doi:10.1242/dev.204964)
Supplement: Supplementary information [file develop-153-204964-s1.pdf]

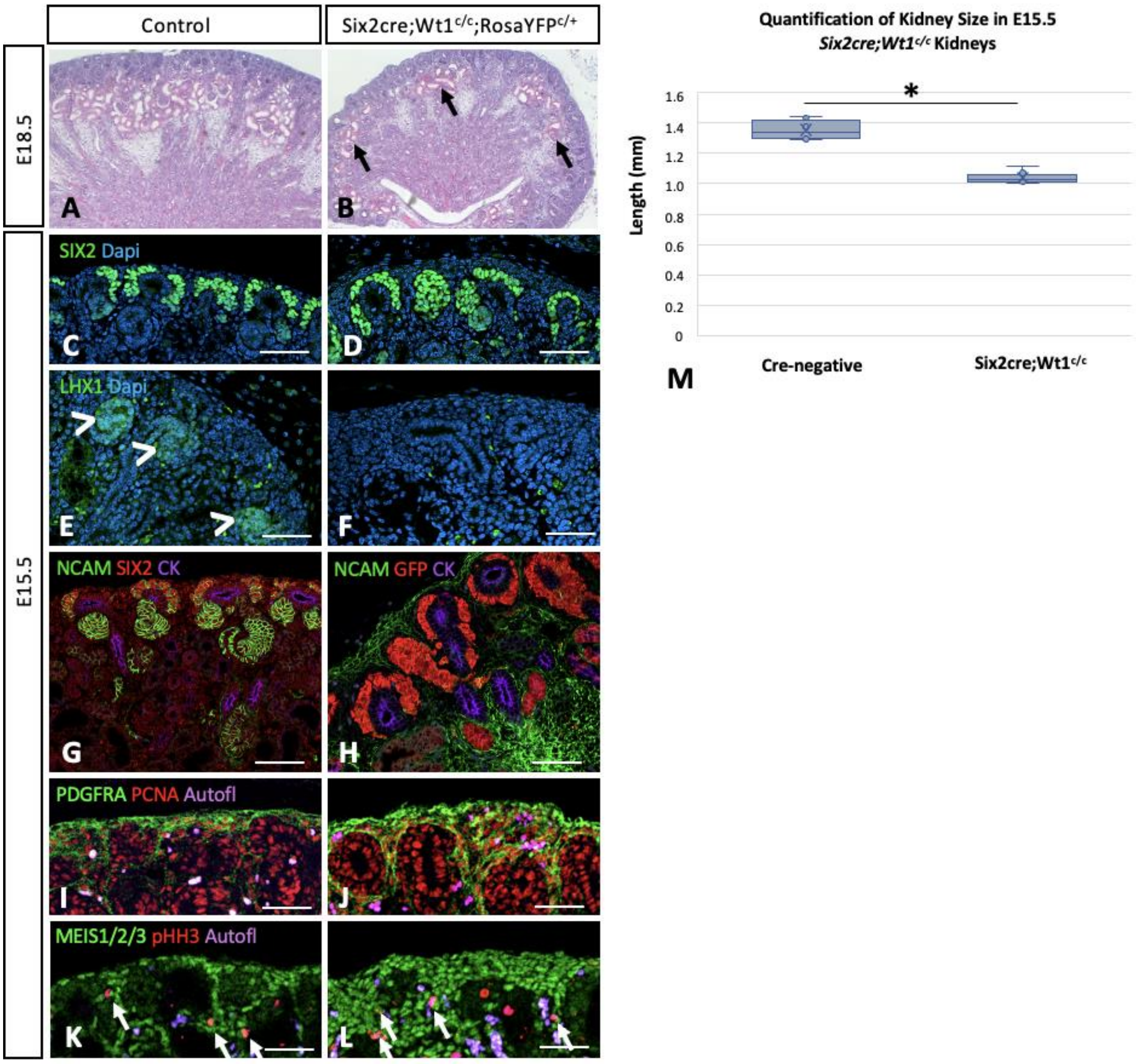

Fig. S1. Characterization of the *Six2cre;Wt1$^{c/c}$* mutant mouse model. (A-B) Histology of E18.5 cre-negative control kidneys (A) is shown in comparison to *Six2cre;Wt1$^{c/c}$* mutant kidneys with defects in nephrogenesis, though some proximal tubules and glomeruli are observed (B, arrows) presumably due to incomplete efficiency of the *Six2cre* model. (C-L) Immunofluorescence of E15.5 control and mutant kidneys show NPCS (labeled with SIX2) maintained in mutant kidneys (D) with early differentiating nephron structures (labeled with LHX1, arrowheads) and NCAM (G) lost in mutant kidneys (F and H, respectively), with mutant NPCs additionally showing loss of NCAM expression. Inclusion of a *Rosa26^{EYFP}* reporter (labeled with GFP antibody) confirms that *Six2cre* specifically targets the NPC lineage with no recombination observed in the stroma as expected (H). Evaluation of proliferation markers including proliferating cell nuclear antigen (PCNA) and pHH3 (phospho-histone H3) suggest proliferation of nephrogenic zone stroma of mutant kidneys (J and L, respectively). M) Quantification of kidney length confirms decrease size in *Six2cre;Wt1$^{c/c}$* mutant kidneys vs littermate cre-negative controls (asterisk denotes unpaired t-test p value of 0.002).

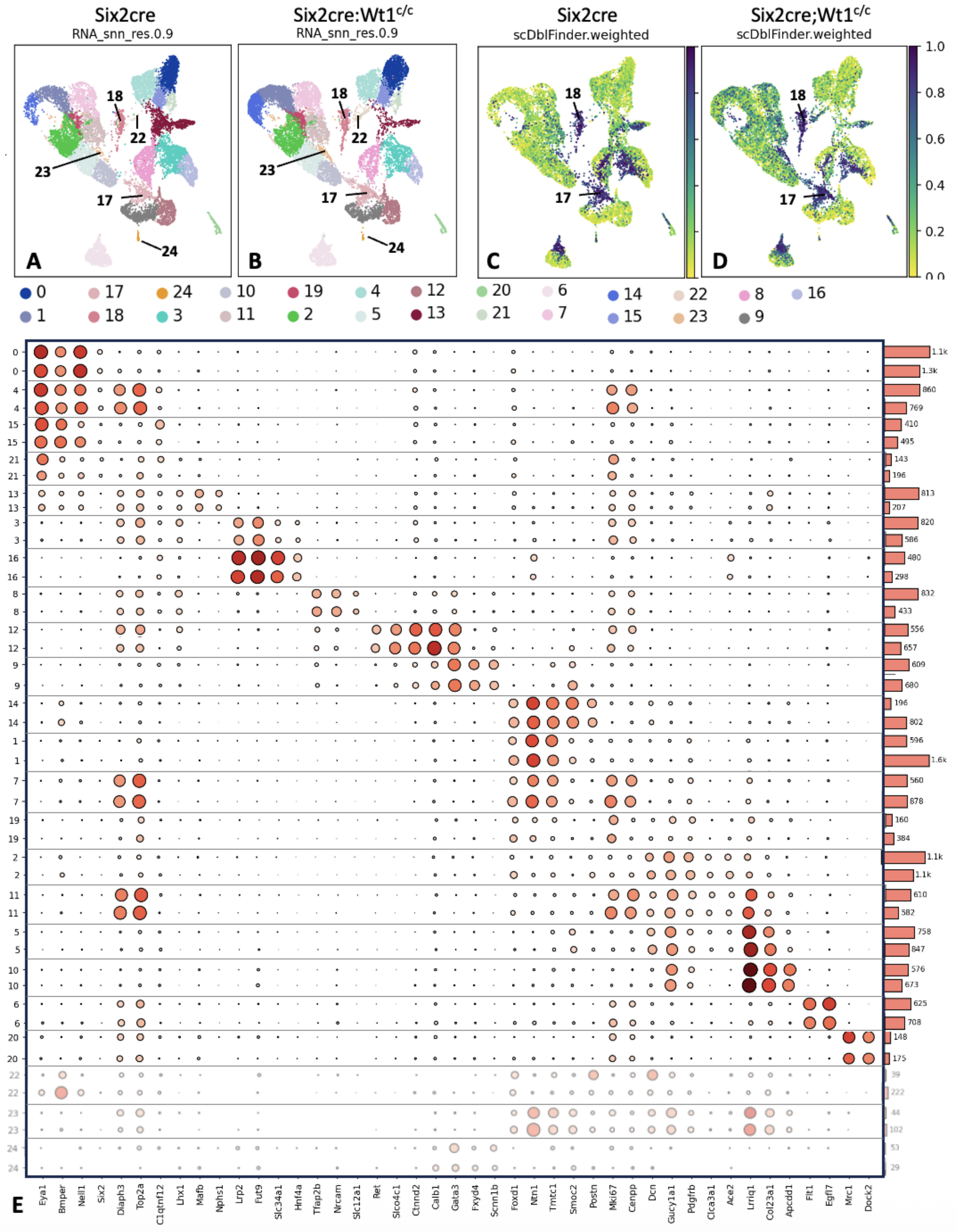

Fig. S2. Unsupervised clustering of snRNA-seq from E15.5 control (*Six2cre*) and mutant (*Six2cre;Wt1$^{c/c}$*) kidneys. A-B) A total of 25 clusters were identified by unsupervised clustering in both control and mutant kidneys. C-D) Clusters 17 and 18 showed high doublet scores in both the control and mutant samples and thus were not included in further analyses, with additional concern for high percentages of doublets in clusters 22, 23, and 24. E) Informed cell clustering was compared for each sample, with the first row from control kidneys and second row from mutant kidneys, so that the expression profile could be directly compared for each identified cell type. Given the lack of marker specificity and low number of nuclei captured in clusters 22, 23, and 24, these three additional clusters were also excluded from further analyses.

Development • Supplementary information

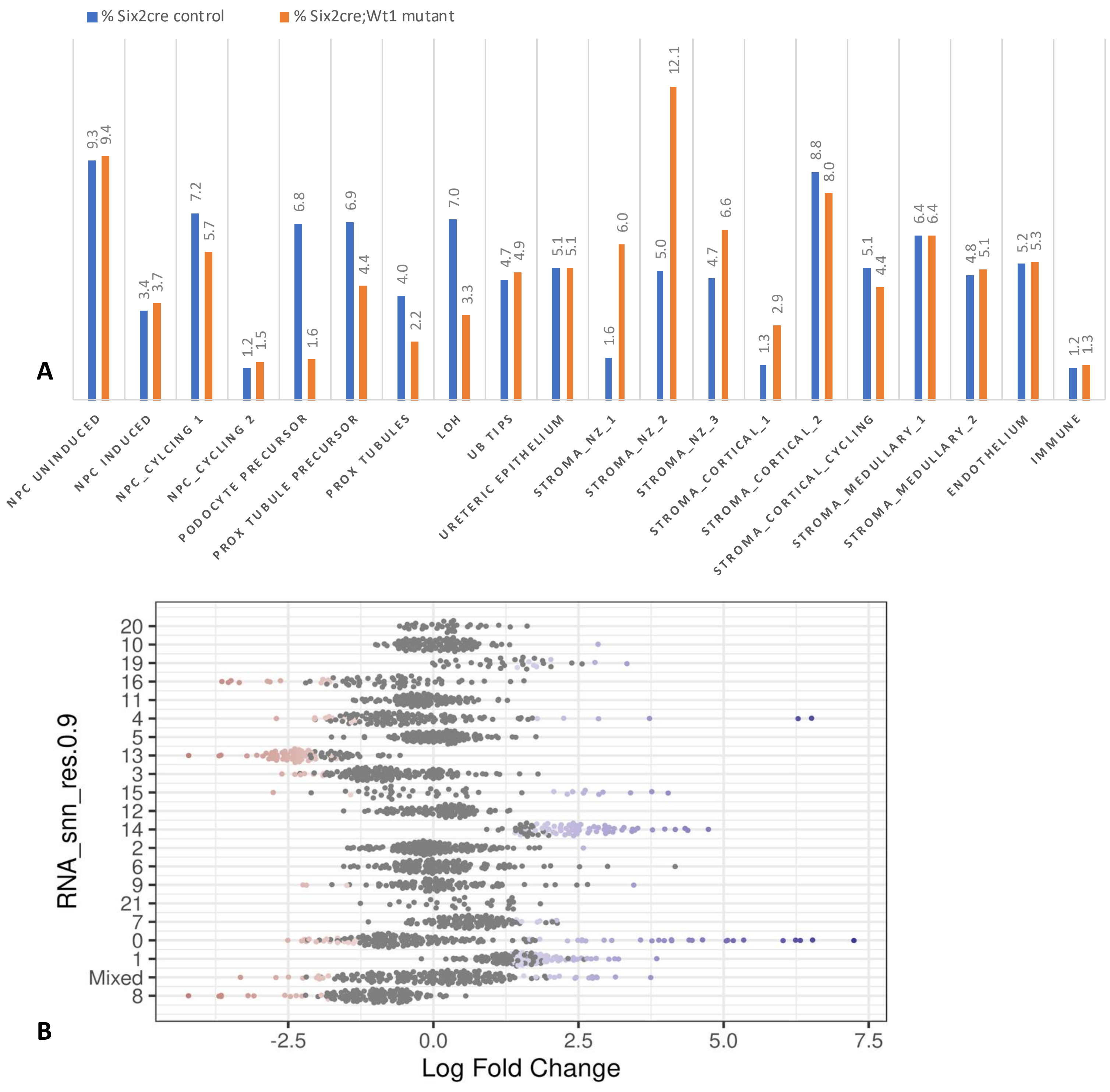

Fig. S3. Comparison of cell types captured in E15.5 control (*Six2cre*) and mutant (*Six2cre;Wt1*$^{c/c}$) snRNA-seq. A) The percent of isolated nuclei of each identified cell type in the 20 clusters were compared for 11,906 nuclei from control (*Six2cre*) kidneys and 13,291 nuclei from mutant (*Six2cre;Wt1*$^{c/c}$) kidneys generated from three separate sequencing experiments using paired kidneys from a total of six embryos. Mutant kidneys showed decreased numbers of differentiated nephron components, with fewer podocyte/ podocyte precursors (i.e., 1.6% vs 6.8%), proximal tubules (i.e., 2.2% vs 4.0%), and loop of Henle (LoH; i.e., 3.3% vs 7.0%) with increased numbers of stromal progenitor nuclei from all three subclusters of the *Foxd1*+ stroma (i.e., 6.0% vs 1.6% for cluster 14, 12.1% vs 5.0% for cluster 1, and 6.6% vs 4.7% for cluster 7). B) Differential abundance testing was conducted to evaluate for statistical differences the cell types captured between control and mutant samples, with p-values shown in Table S1.

Development • Supplementary information

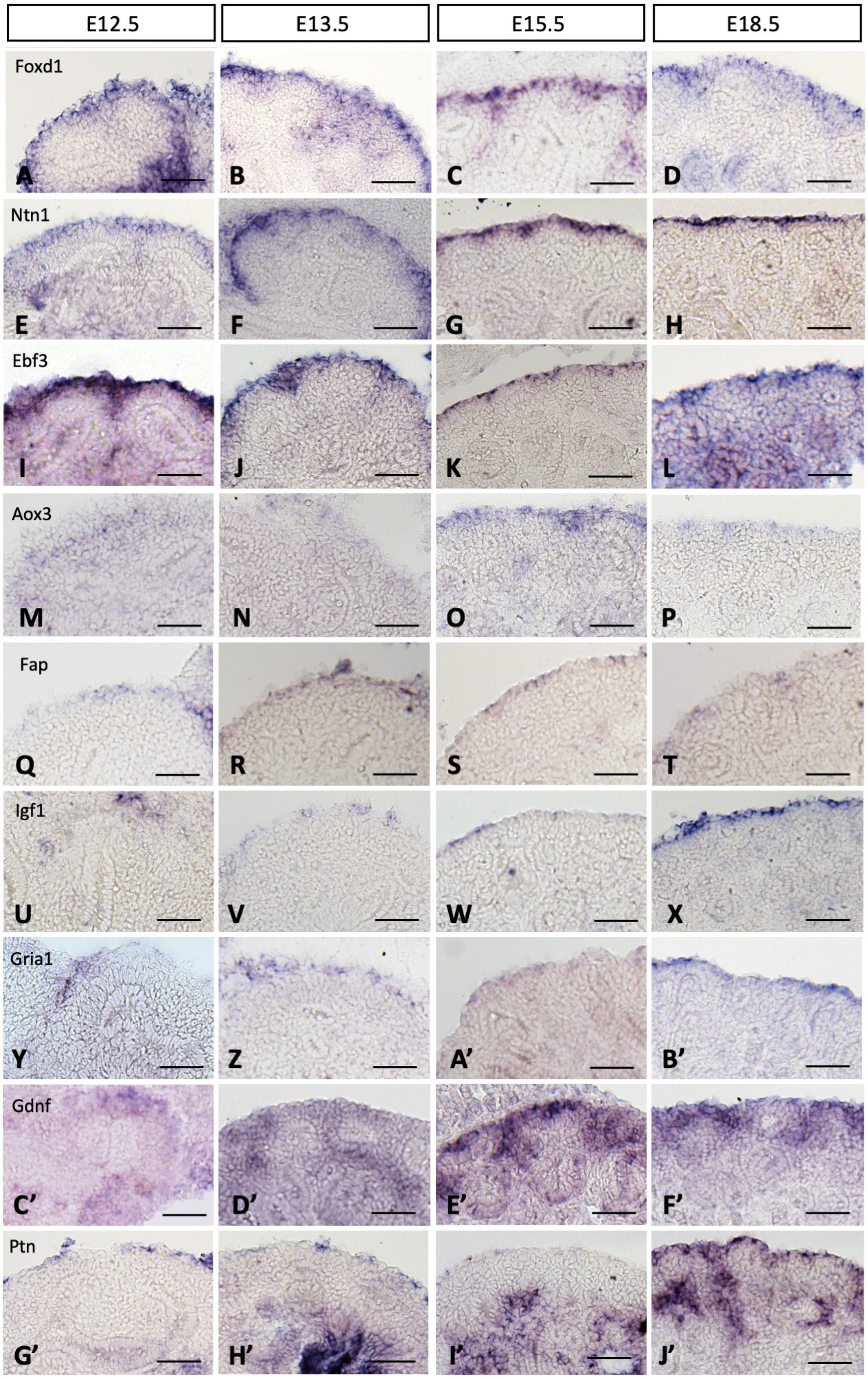

Fig. S4. Expression of stromal progenitor cell markers throughout time points of normal kidney development. Identified markers of the *Foxd1*+ stromal progenitor population from snRNA-seq were evaluated at E12.5, E13.5, E15.5, and E18.5 in control kidneys.

Development • Supplementary information

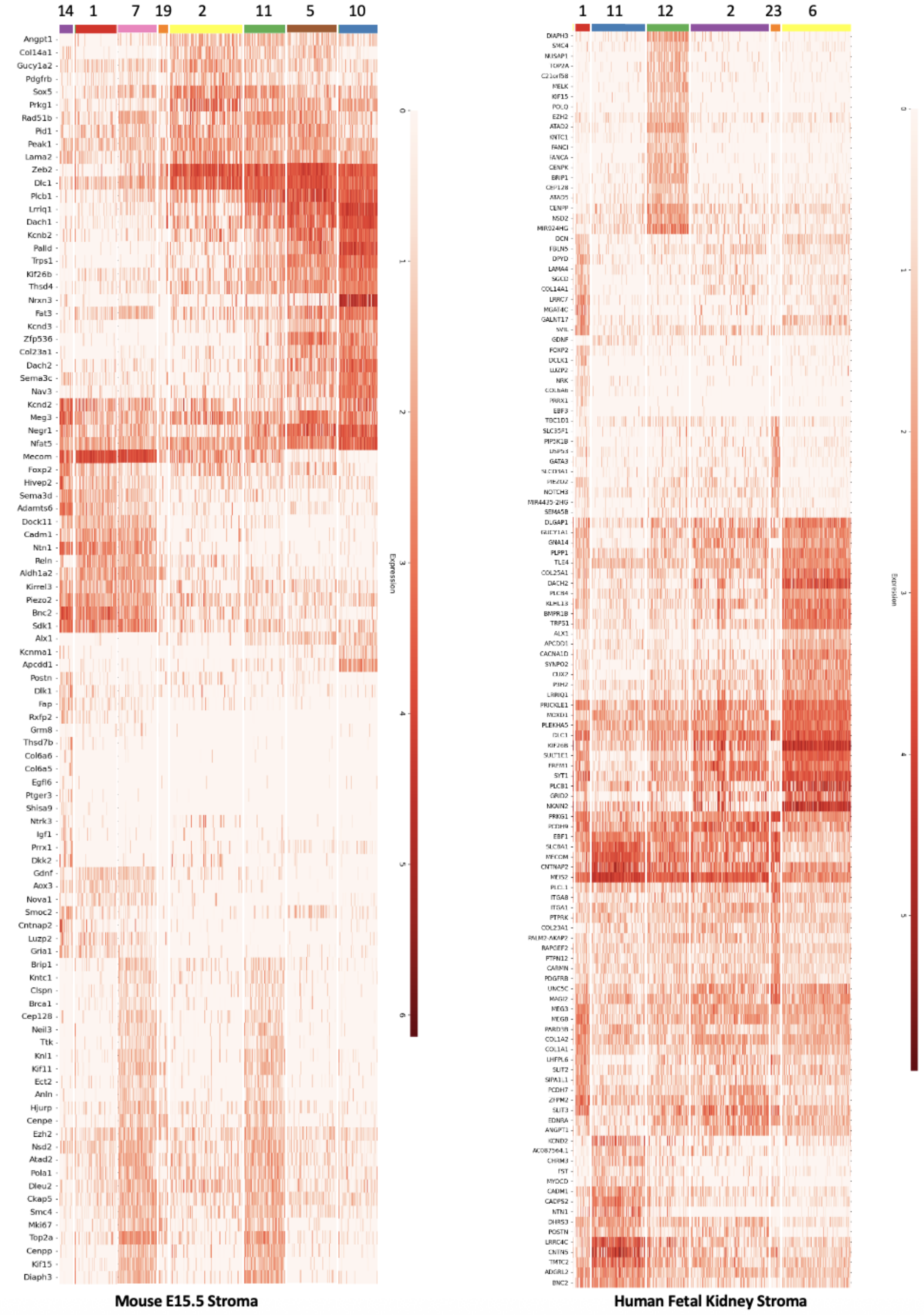

Fig. S5. Heatmap of stromal clusters identified in murine and human fetal kidneys. Analysis of snRNA-seq of murine and human fetal kidneys identified six subclusters of stroma, with transcriptional profiles of marker genes and top DEGs shown via heatmap.

Development • Supplementary information

Table S1. E15.5 mouse con_Marker genes.xlsx

Available for download at
https://journals.biologists.com/dev/article-lookup/doi/10.1242/dev.204964#supplementary-data

Table S2. E15.5 mouse con_DEG_NZ stroma.xlsx

Available for download at
https://journals.biologists.com/dev/article-lookup/doi/10.1242/dev.204964#supplementary-data

Table S3. Cell types captured in control (*Six2cre*) and mutant (Six2cre;Wt1$^{c/c}$) snRNA-seq

Cluster	Cell type	Six2cre nuclei #	Six2cre;Wt1$^{c/c}$ nuclei #	Six2cre % nuclei	Six2cre;Wt1$^{c/c}$ % nuclei	Adj P value
0	NPC uninduced	1102	1254	9.3	9.4	NS
15	NPC induced	410	495	3.4	3.7	NS
4	NPC_cycling 1	860	760	7.2	5.7	NS
21	NPC_cycling 2	143	196	1.2	1.5	NS
13	Podocyte precursor	813	207	6.8	1.6	**1.7E-46**
3	Prox tubule precursor	820	586	6.9	4.4	**2.4E-17**
16	Prox tubules	480	298	4.0	2.2	**6.1E-8**
8	LoH precursor	832	433	7.0	3.3	**2.8E-31**
12	UB tips	556	657	4.7	4.9	NS
9	Ureteric epithelium	609	680	5.1	5.1	NS
14	Stroma_NZ_1	196	802	1.6	6.0	**2.1E-39**
1	Stroma_NZ_2	596	1613	5.0	12.1	**3.3E-63**
7	Stroma_NZ_3	560	878	4.7	6.6	**3.3E-15**
19	Stroma_cortical_1	160	384	1.3	2.9	**9.9E-10**
2	Stroma_cortical_2	1052	1063	8.8	8.0	NS
11	Stroma_cortical_cycling	610	582	5.1	4.4	NS
5	Stroma_medullary_1	758	847	6.4	6.4	NS
10	Stroma_medullary_2	576	673	4.8	5.1	NS
6	Endothelium	625	708	5.2	5.3	NS
20	Immune	148	175	1.2	1.3	NS
Total		11906	13291			

Table S4. E15.5 mouse con_vs_mut_DEG_NZ stroma.xlsx

Available for download at
https://journals.biologists.com/dev/article-lookup/doi/10.1242/dev.204964#supplementary-data

Table S5. Human fetal kidney_Marker genes.xlsx

Available for download at
https://journals.biologists.com/dev/article-lookup/doi/10.1242/dev.204964#supplementary-data

Development • Supplementary information
